# Broadly neutralizing antibodies and vaccine design against HIV-1 infection

**DOI:** 10.1007/s11684-019-0721-9

**Published:** 2019-12-19

**Authors:** Qian Wang, Linqi Zhang

**Affiliations:** Comprehensive AIDS Research Center, Beijing Advanced Innovation Center for Structural Biology, Collaborative Innovation Center for Diagnosis and Treatment of Infectious Diseases, Department of Basic Medical Sciences, School of Medicine, Tsinghua University, Beijing, 100084 China

**Keywords:** HIV-1, broadly neutralizing antibodies, Env conformational states, vaccine design, SOSIP

## Abstract

Remarkable progress has been achieved for prophylactic and therapeutic interventions against human immunodeficiency virus type I (HIV-1) through antiretroviral therapy. However, vaccine development has remained challenging. Recent discoveries in broadly neutralizing monoclonal antibodies (bNAbs) has led to the development of multiple novel vaccine approaches for inducing bNAbs-like antibody response. Structural and dynamic studies revealed several vulnerable sites and states of the HIV-1 envelop glycoprotein (Env) during infection. Our review aims to highlight these discoveries and rejuvenate our endeavor in HIV-1 vaccine design and development.

## Introduction

Human immunodeficiency virus type I (HIV-1) is the cause of the global epidemic of acquired immunodeficiency syndrome (AIDS) [1,2]. Since its discovery in the early 1980s, HIV-1 has infected more than 70 million individuals and caused 35 millions deaths worldwide [3]. Approximately 1.2 million of all infected cases originated from China [4,5]. Antiretroviral therapy can achieve sustained viral suppression, extend the life span of infected individuals, and reduce HIV transmission [6,7]. However, the establishment and persistence of a latent viral reservoir in infected patients pose a huge barrier for eliminating the virus and ultimately curing this disease [8,9].

A safe and effective vaccine is a remarkable prospect for blocking HIV-1 infection. However, the high degrees of sequence and structural diversity within and between infected individuals enable viral escape from immune recognition. To date, traditional vaccine approaches have failed to overcome HIV-1 diversity despite the remarkable success against a large array of pathogens with minimal variability [10]. Out of over 100 vaccine trials, only the RV144 trial has achieved positive yet moderate protection (31.2%) [11]. Therefore, a sophisticated and rational approach is needed to develop protective vaccines against HIV-1. The major focus of vaccine design approaches is the envelope glycoprotein (Env) because it is the sole target for broadly neutralizing antibodies (bNAbs) [12,13]. Technical advancements in antibody technology have facilated identification of a new generation of bNAbs targeting various regions of HIV-1 Env [13–15]. Structure and function studies on specific epitope and conformational states recognized by these bNAbs have provided unprecedented opportunities for vaccine design to induce bNAb-like antibody response.

## HIV-1 bNAbs

HIV-1 bNAbs can neutralize the majority of HIV-1 strains from diverse genetic and geographic backgrounds. bNAbs are broadly classified into two major generations on the basis of their neutralization activity and the time of their initial isolation [16]. First-generation bNAbs were isolated in the 1990s and have exhibited limited neutralization potency and breadth. However, new potent and broad neutralization antibodies have been isolated since 2009 and now represent second-generation bNAbs.

## First-generation bNAbs

First-generation bNAbs include b12, 2G12, 2F5, and 4E10, which were isolated through phage surface display and Epstein–Barr virus transformation of B cells derived from HIV-1-infected patients [17–20]. The molecular characterization of these antibodies helped identify several vulnerable sites on gp120 and gp41 of HIV-1 Env. These sites include the CD4 binding site (CD4bs) targeted by b12 [17,21], OD-glycans by 2G12 [18], and membrane proximal external region (MPER) of gp41 by 4E10 and 2F5 [19,20]. Although these antibodies are relatively weak in breadth and potency, the passive transfer of high doses of b12, 2G12, 2F5, and 4E10 produced impressive levels of protection against simian–human immunodeficiency viruses (SHIVs) in rhesus macaques [22–27]. Given that b12 is derived from a phage display antibody, its heavy and light chain combinations are considered unnatural [17]. 2G12 has an atypical structure, especially in the combining sites of the antigen-binding fragment (Fab) [18,28]. 2F5 and 4E10 are polyreactive [29,30]. Despite encouraging results in animal models, clinical trial data revealed that the combinations of these first-generation bnAbs failed to effectively suppress viremia in humans [31].

## Second-generation bNAbs

Approximately 10%–25% of HIV-1-infected individuals exhibit broad and potent neutralizing activities against a diverse panel of circulating virus strains [32–37]. Cloning of antigen-specific antibodies from naturally occurring B cells has helped obtain dozens of second-generation bNAbs from HIV-1-infected individuals [13,14]. Sequence mapping and structural analysis of antibody and antigen complexes have led to the identification of several vulnerable targets on HIV-1 Envs, such as N-glycan-associated epitopes on the V1/V2 (PG9, PGT145) and V3 regions (PGT121, 10-1074), CD4bs (VRC01, N6, 3BNC117), gp120/gp41 interface (35O22, 8ANC195), fusion peptide (VRC34.01, ACS202), silent face center (VRC-PG05, SF12), and MPER on gp41 (10E8). The specific epitopes targeted by each of these bNAbs and their unique features are summarized in Table 1 and Fig. 1. Recent developments in the use of bnAbs for HIV-1 prevention and treatment are outlined in Fig. 2 [38,39].

### V1V2

The apex V1V2 of Env is masked by glycans and variable loops [40,41]. Structural analysis shows that V1V2-specific antibodies, such as PG9, PG16, CH01-04, PGT141-145, PGDM1400-1412, and CAP256-VRC26.01-33, utilize the long, anionic complementarity-determining region 3 loops of heavy chain (CDRH3) to penetrate the glycan shield and interact with a quaternary epitope formed by V1V2 and N156/N160 glycans [42–46].

### V3-glycan

Glycan-V3-directed bNAbs, such as PGT121- and PGT128-like antibodies, come in contact with a high-mannose patch at/near N332 in the V3 region through protruding loop regions with various angles [44,47]. Common examples in this category include PGT121-like antibodies, which are bound to the base, and PGT128-like antibodies, which interact with the C-terminal end of the V3 loop [47–49]. These antibodies prevent HIV-1 infection by interfering with CD4 and CCR5 co-receptor binding capabilities [48,50].

### CD4bs

CD4 molecules on the cell surface serve as primary binding targets for HIV-1 Env gp120 [51,52]. CD4bs is functionally conserved and structurally recessed on gp120, surrounded by multiple glycans. Despite its recessed nature, CD4bs is accessible to CD4 molecules and various CD4bs-specific antibodies [53]. CD4bs exhibits a good amount of heterogeneity in its sequence and structure among HIV-1 strains but can induce the largest number of known bNAbs during infection [54]. Several CD4bs antibodies neutralize over 90% of HIV-1 strains, including VRC01, VRC07, N6, 3BNC117, and N49P [55–58]. One of the most potent ones is the recently identified N49P antibody that can neutralize nearly 100% of a global panel comprising 117 pseudoviruses [55]. X-ray crystallographic analysis showed that N49P7 could bypass the CD4bs Phe43 cavity and reach deeply into the highly conserved residues of layer 3 of the gp120 inner domain. These unique features may explain the extreme potency and breadth of this antibody. In addition, these CD4bs bNAbs share some common features in mode of action and compostion of variable domains such as mimicking CD4 to achieve their known distinguished breadth [59], using VH1-2 germline origin genes of the heavy chain, and having a short five-amino-acid complementarity determining region 3 of the light chain (CDR L3) [55–59]. However, many resistant strains are against this class of antibodies in naturally infected individuals [60]. Treatment strategies based on CD4bs antibodies must overcome such resistance to achieve optimal clinical outcomes.

### MPER

MP ER-specific antibody 10E8, which was isolated by Huang *et al.* in 2012, neutralized approximately 98% of tested viruses and had a geometric mean $IC_{50}$ value of 0.352 μg/mL [61]. Different from the first-generation MPER-directed bNAbs 2F5 and 4E10, 10E8 is not

**Table 1** Overview of characteristics of representative second-generation bNAbs

| Ab | Epitope | $IC_{50}$ geometric mean (μg/mL) | Neutralization breadth (%) | Number of viruses tested | Clinical bNAb development | VH mutation (nt %) | CDRH3 length (aa) | Polyreactivity | Preferentially bind | References (PMID) |
|---|---|---|---|---|---|---|---|---|---|---|
| PGDM1400 | V1V2 | 0.003 | 83 | 106 | Yes | 24.3 | 34 | No | NA | 25422458 |
| PG9 | V1V2 | 0.220 | 79 | 162 | No | 13 | 28 | No | State 1 Env | 19729618, 30971821 |
| PGT145 | V1V2 | 0.290 | 78 | 162 | No | 18 | 31 | No | State 1 Env | 21849977, 25298114 |
| PG16 | V1V2 | 0.150 | 73 | 162 | No | 12 | 28 | No | State 1 Env | 19729618, 25298114 |
| VRC26.25 | V1V2 | 0.003 | 63 | 200 | Yes | 12 | 36 | No | NA | 26468542 |
| PGT128 | V3 glycan | 0.020 | 72 | 162 | No | 19 | 19 | No | State 1 Env | 21849977, 25298114 |
| PGT121 | V3 glycan | 0.030 | 70 | 162 | Yes | 17 | 24 | No | NA | 21849977 |
| 10-1074 | V3 glycan | 0.022 | 66 | 178 | Yes | 26.7 | 24 | NA | State 1 Env | 23115339, 30971821 |
| BG18 | V3 glycan | 0.032 | 62 | 119 | No | 21.2 | 21 | NA | NA | 28100831 |
| N49-P7 | CD4bs | 0.100 | 100 | 117 | No | 24.1 | 21 | NA | NA | 29731169 |
| N6 | CD4bs | 0.038 | 98 | 181 | Yes | 31 | 15 | No | NA | 27851912 |
| VRC07-523 | CD4bs | 0.039 | 96 | 179 | Yes | 23.5 | 18 | Yes | NA | 25142607 |
| VRC01 | CD4bs | 0.369 | 90 | 208 | Yes | 32 | 14 | Yes | State 1 Env | 20616233, 25298114 |
| 3BNC117 | CD4bs | 0.126 | 88 | 120 | Yes | 29.7 | 12 | Yes | State 1 Env | 21764753, 30971821 |
| NIH45-46 | CD4bs | 0.098 | 86 | 120 | No | 32.8 | 18 | Yes | NA | 21764753 |
| DH511.11P | MPER | 0.630 | 100 | 210 | No | 10.6 | 23 | No | NA | 28783671 |
| 10E8 | MPER | 0.352 | 98 | 180 | Yes | 21 | 22 | No | NA | 23151583 |
| 8ANC195 | GP120/GP41 interface | 1.189 | 68 | 120 | No | 28 | 22 | Yes | NA | 21764753 |
| 35O22 | GP120/GP41 interface | 0.033 | 62 | 181 | No | 35 | 16 | Yes | NA | 25186731 |
| PGT151 | Fusion peptide | 0.008 | 66 | 117 | No | 20 | 28 | No | State 2 Env | 24768347, 30971821 |
| VRC34.01 | Fusion peptide | 0.320 | 49 | 208 | No | 15 | 13 | NA | NA | 27174988 |
| ACS202 | Fusion peptide | 0.142 | 45 | 75 | No | 16.1 | 22 | Yes | NA | 27841852 |
| SF12 | Silent face center | 0.200 | 62 | 119 | No | 17 | 23 | No | NA | 31126879 |
| VRC-PG05 | Silent face center | 0.800 | 27 | 208 | No | 9 | 17 | NA | NA | 29548671 |

Abbreviations: SHM, somatic hypermutation; NA, not available.

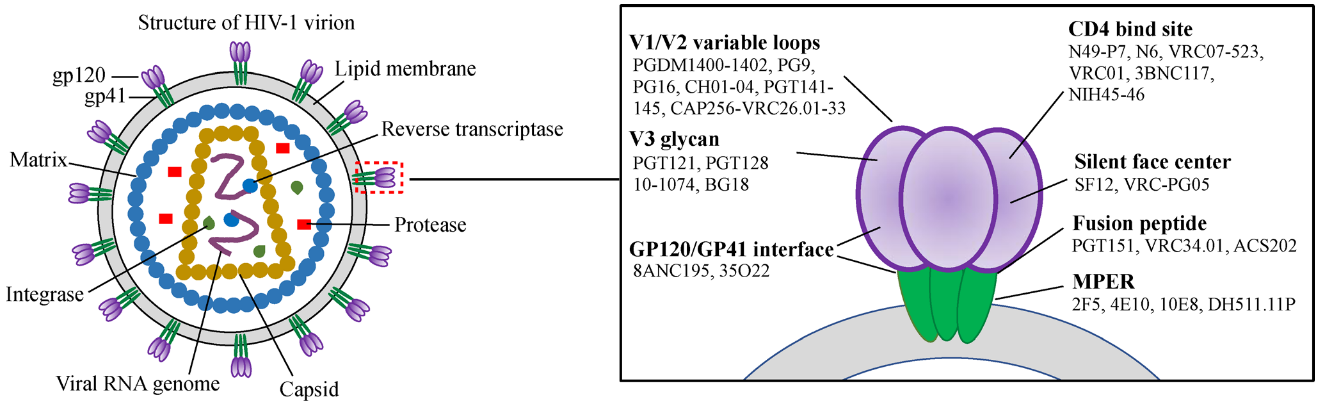

**Fig. 1** Schematic diagram of HIV-1 and epitopes for bNAbs on trimeric HIV envelope spike glycoproteins.

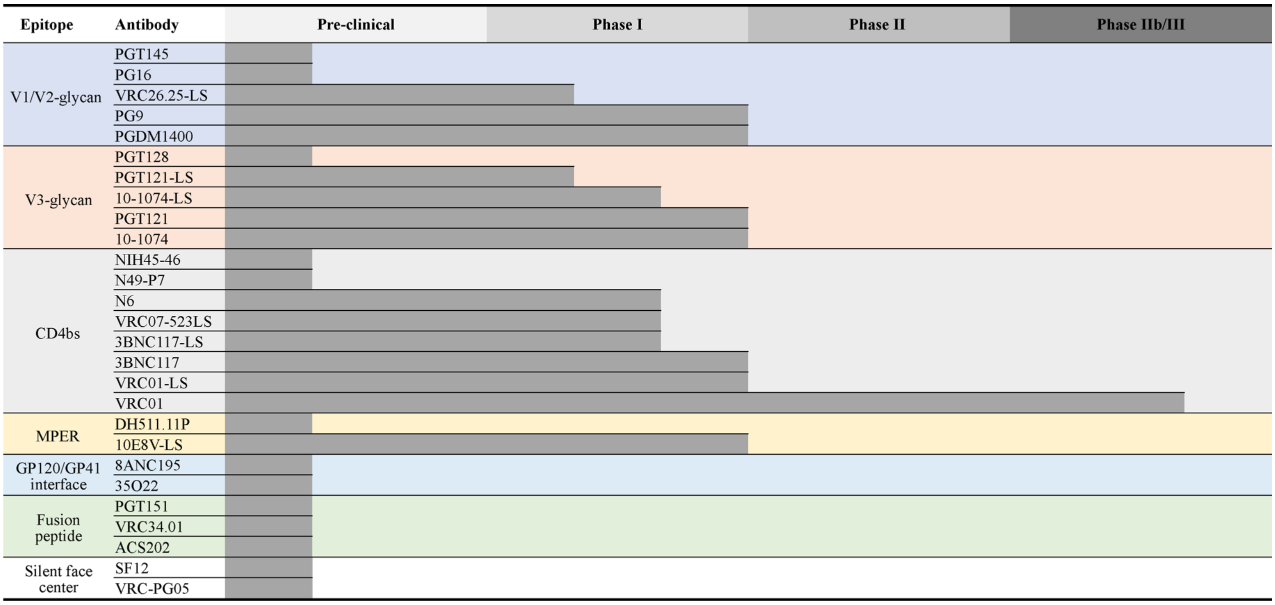

**Fig. 2** Current status of bNAb development in preclinical and clinical studies.

phospholipid- or auto-reactive and recognizes the highly conserved helix at the C-terminal half of the MPER region that is proximal to the transmembrane region. The development of these antibodies and detailed potential strategies of inducing 10E8-like antibody through vaccination have been recently highlighted. A multimeric founder MPER can stimulate B cells bearing the unmutated common ancestor of the 4E10-like antibody VRC42. Therefore, immunogens containing the specific founder MPER can be utilized to stimulate naive B cells to initiate 4E10- and 10E8-like antibodies [62].

## GP120/GP41 interface

35O22, which targets the gp120/gp41 interface, was isolated by Huang *et al.* in 2014 [63]. This antibody neutralized 62% of 181 tested pseudoviruses with a geometric mean $IC_{50}$ value of 0.033 μg/mL. Similar to 35O22, 8ANC195 also recognized the subunit interface of the prefusion-closed conformation of Env [64]. Both of these antibodies are immune to the conformational change of Env triggered by CD4 molecules, while other bnAbs are not. 8ANC195 can even bind the gp120 monomer in the absence of gp41 [65].

## Fusion peptide

The 15 to 20 hydrophobic residues, termed fusion peptide, at the N terminus of the gp41 subunit are another vulnerable site recognized by bNAbs such as VRC34.01,

PGT151, and ACS202 [66–68]. The residual sequences of fusion peptide are highly conserved and strain-specific [66]. Interestingly, ACS202 shows broadly neutralization activity against recombinant CRF01_AE viruses, while VRC34.01 and PGT151 fails [66–68]. VRC34.01, PGT151, and ACS202 can approach fusion peptide from various angles and function in multiple conformations of fusion peptide [69]. However, the generation of these antibodies during infection and their mechanism of neutralization remain largely unknown. Given their relatively conserved nature among circulating HIV-1, fusion peptided represents a promising vaccine candidate [70,71].

### Silent face

The silent face represents a relatively new target for generating bNAbs. This site is not substantially affected by glycan alteration and therefore can maintain sensitivity to antibody recognition and neutralization. However, the potency and breadth of this class of antibodies are generally less than those of the abovementioned bNAbs. For example, the VRC-PG05-like antibody, which targets the center of the silent face, shows ~0.8 μg/mL (geometric mean $IC_{50}$s) potency and 27% breadth [72]. In comparison, the recently isolated SF12 antibody shows greater potency of ~0.20 μg/mL (geometric mean $IC_{50}$s) and 62% breadth [73]. Nevertheless, these two antibodies highlight the silent face epitope as a new and potential target for vaccine-directed response.

## Common features of HIV-1 bNAbs

HIV-1 bNAbs can neutralize diverse HIV-1 strains that possess one or more unusual traits, such as uncommon recombination patterns, extensive somatic hypermutation (SHM), long IgH CDR3s, and self- or polyreactive nature [74,75]. The high levels of mutation and long CDRH3 are the result of multiple rounds of affinity maturation and selection through interaction with evolving Envs in the germinal center. Virus escape sometimes induces the development of neutralizing antibodies in an unusual, co-evolutionary manner [62,76,77]. This phenomenon highlights the complexity in the process and pattern of bNAb development. Not all Envs or conformational states are equal to binding and neutralization by bNAbs. Most bNAbs are prone to binding conformational state 1 of Envs, indicating conformational vulnerability in addition to the epitope vulnerability of the Env of HIV-1 [78].

### High SHM

HIV-1 bNAbs directed at the Env of the virus are rarely developed in acutely infected individuals and typically emerge only after several years of infection [79,80]. HIV-1 bNAbs have high levels of SHM as a result of multiple rounds of antigen recognition and stimulation [15,81]. For example, manifold improvement in the neutralizing potency and breadth of HIV-1 bNAbs has been observed after 40–100 gene somatic mutations in the heavy chain. In constrast, most human antibodies often carry 10–20 gene mutations [82,83]. Amino acid variation greater than 30%–40% has been observed in the heavy-chain bNAbs targeting CD4bs [15]. In addition to the expected region of CDRs, mutations also appear in the framework regions (FWRs) of these bNAbs [84]. A FWR is typically less tolerant of mutations because of the potential structural destruction of the variable domain.

In addition to amino acid substitutions, HIV-1 bNAbs present a large number of length polymorphisms, such as insertion and deletions, which could be up to sevenfold higher than those of most human antibodies [85]. Insertions and deletions could be observed in the heavy and light chains of CDRs and FWRs [85]. When HIV-1 bNAbs are reverted to their germline ancestor sequences, their neutralization and binding abilities to most HIV-1 Envs are reduced and even completely lost [86].

The exact mechanism for the high mutation of HIV-1 bNAbs remains unclear. One hypothesis is that only some specific HIV-1 Envs can activate and trigger germline antibody-expressing B cells. The activated B cells are further stimulated by the continuously evolving and mutating Envs during infection. This phenomenon persists until the antibody gene has a sufficient and appropriate number of mutations that can recognize diverse Env variants. Another hypothesis is that the B cells expressing germline antibodies first engage with non-HIV-1 antigen and then develop cross-reactivity with HIV-1 Envs through a series of mutations [87]. Both hypotheses have some supporting evidence, although they are not necessarily mutually exclusive.

### Long CDRH3

HIV-1 bNAbs generally have long CDRH3 that enables the penetration of the glycan shield of the envelope [15]. Most human antibody CDRH3s range from 8 to 16 amino acids in length, whereas HIV-1-specific antibodies average at approximately 17.8 amino acids. In particular, HIV-1 bNAbs have CDRH3 with an average length of 20.9 amino acids [81]. For example, the CDRH3 of the V2-glycan-specific antibody VRC26-CAP256 is as long as 36 amino acids [46]. Such long HCDR3 of HIV-1 bNAbs indicates major challenges for HIV-1 B cell vaccine development. To overcome this barrier, some novel vaccine strategies have been proposed starting with triggering the germline ancestors followed by boosting various intermediate for the ultimate induction of bNAbs in human [88].

### Polyreactivity

Polyreactivity is a common but unusual characteristic of HIV-1 bNAbs that allow promiscuous binding to unrelated self- and/or foreign-antigens with a fairly low affinity. Approximately 55%–75% of antibodies produced by early immature B cells in the nascent repertoire exhibit this feature. Polyreactive bNAbs are mostly removed from the system, although approximately 5% of mature B cells retain the ability to produce such bNAbs [89]. Approximately 70% of HIV-1 Env-specific antibodies are polyreactive [90]. Therefore, the relatively low frequency of HIV-1 bNAbs in natural infection may be due to the clearance and deletion of poly- and auto-reactive B cell clones.

The exact mechanism for the high polyreactivity in HIV-1 bNAbs is unclear. Evidence suggests that polyreactivity may help increase antibody affinity and avidity for Envs through binding to a second non-HIV-1 antigen on the virion. The density of Env spikes alone is extremely low for the bivalent binding of HIV-1 bNAbs. The estimated density of Env on a mature HIV-1 virion is approximately 14 per virion [91]. For example, MPER-specific antibodies 2F5 and 4E10 bind to gp41 and viral membrane [29,30]. Antibody b12 binds to CD4bs and to ribonucleoprotein and double-stranded DNA [17]. Other CD4b antibodies (12A12, NIH45-46, CH103, CH104, and CH106) and V1V2 antibodies (CH03) also exhibit varying degrees of polyreactivity [74].

### Preferential binding of bNAbs to conformational state 1 of Env

HIV-1 Env on the surface of virion spontaneously transits through at least three different conformational states: states 1, 2, and 3 [92,93]. All these states are independent of the ligand interaction status with CD4 molecules. After interaction and binding to CD4, a particular bias for a certain state of the Env occurs. State 1 Env predominantly stays in a “closed” conformation that corresponds to the CD4-unbound prefusion state; state 2 is an intermediate state with partially open conformation [94,95]. Binding to CD4 and modification of Env residues help drive the Env transitioning into this state [94–97]. State 3 represents an “open” conformation ready for fusion, having been formed and stabilized by CD4 molecules and 17b/co-receptor [94,98].

Recent studies on the dynamics of Env on virion surfaces show that most bNAbs prefer a certain conformational state for binding. For example, many bNAbs favor conformational state 1 over state 2 or 3 of Envs [78,98]. A dynamic study based on single-molecule fluorescence energy transfer (smFRET) reported a preferred Env state for bNAb binding [78], although the exact structural features and their relationship with the recombinant trimeric structures remain unclear. In addition to undergoing spontaneous structural changes on the virion, Env experiences significant structural alterations during HIV-1 entry. Such alterations expose several vulnerable sites on the Env to various antibody-mediated functions, such as neutralization, ADCC, and ADPC [14]. Many human bNAbs bind to CD4 unbound and prefusion-closed Env, indicating that human bNAbs prefer to bind to conformational state 1 [78,98]. The exception to this preference is the bNAb PGT151, which favors Env at states 2 and 3 [78]. The dynamic nature of Env has certainly added another layer of complexity on top of the known sequence and structural diversity of Env. It may also indicate the existence of more vulnerable conformational state where bNAbs could exert effective roles through direct neutralization or an indirect one through ADCC and ADPC.

## HIV Env conformational states and immunogen design

### Reported dynamics of HIV Env conformational states

Along with crosslinking/mass spectrometry, X-ray crystallography, and cryo-electron microscopy (cryo-EM), the recently well-developed technique of smFRET offers the unique advantage of defining the conformational structure of HIV-1 Env on the surface of the virion. Furthermore, a novel conformational state named state 2A was recently identified, which is largely stabilized by binding to anti-cluster A antibodies (such as A32 and C11), anti-co-receptor binding site antibodies (such as 17b), and small-molecule CD4 mimetics (such as BNM-III-170) [99]. State 2A is asymmetric and exists when the Env is partially open. State 2A Env mediates ADCC function, whereas state 1 facilitates bNAbs neutralization [78,98,99].

### Current updates on HIV conformational states and vaccine candidates

The existence of bNAbs shows that the natural B cell response can generate protective neutralizing antibodies against HIV-1 infection. Designing an effective vaccine that induces cross-reactive antibodies is of paramont interest in the HIV-1 field. Among the present strategies, the most popular one involves generating a soluble trimeric form of Env to mimic the native Env on the surface of virion. The first and best studied Env trimer is BG505 SOSIP.664, which contains a disulfide bond (SOS), an I559P mutation in gp41 (IP), and a truncation at position 664 to stabilize the trimeric form [100]. Antigenicity studies showed that the BG505 SOSIP.664 trimer displays epitopes that are recognized by bNAbs but not by nonbNAbs which closely resemble the conformational structure of the native envelope [100–102]. Additional forms of soluble trimers, such as native flexibly linked (NFL) [103,104] and uncleaved prefusion-optimized (UFO) [105] are also being designed and evaluated. The design of trimer Envs take advantage of the structural information recently obtained through high-resolution X-ray crystallography and cryo-EM [104,106–109]. Many other forms of envelope, such as gp120 monomers, gp120-monomeric core (such as RSC3, 2CC, and 426c core), germline-targeted proteins (such as eOD-GT proteins), epitope-specific scaffolds (such as FP-KLH), and epitope-based antigenic domain (such as EAD), are also being designed and evaluated as potential immunogens [70,103,105,110–115]. Although each of these immunogens has its own design features and rationale, the ultimate test of their potential as vaccine, that is, their safety, immunogenicity, and protectivity, depends on human studies.

Preliminary results from animals such as mice, guinea pigs, rabbits, and monkeys immunized with SOSIP.664 failed to elicit satisfactory levels of bNAbs against heterologous tier-2 viruses [116–122]. However, the same immunogen can induce broad and potent serum antibody response in cows and dromedaries, suggesting that the long CDRH3 of antibodies in these animals may provide good genetic bases for generating neutralizing antibodies [123,124]. Translating the findings in cows and dromedaries into vaccine design for humans remains a challenge. Cow antibodies isolated from immunized animals preferably target the conformational state 2 of Env. Further studies are needed to determine whether this preference is related to the nature of cow antibody triggering virus Env conformation state 2 or whether SOSIP.664 intricately induces state 2-preferred antibodies. If the former case applies, then it is a possibility that the cow antibodies would likely trigger Env into unfavored state. If the latter case applies, then the current SOSIP.664 itself might not be the favored conformation given that most human bNAbs prefer binding to state 1 Env. This scenario may provide some explanations for the failure of SOSIP.664 in eliciting neutralizing antibodies in various animal models. Future vaccine design is needed to consider the conformation states of Env.

## Conclusions

Despite the significant progress in the current understanding of HIV-1 bNAbs, the successful induction of bNAbs-like immune responses in humans remains as a daunting challenge. The biggest challenge is our incomplete understanding of how exactly these bNAbs are generated, evolved, and maintained during infection. A recent study used fusion peptide as immunogen in rhesus macaques has yielded some encouraging results where an antibody lineages can neutralize 59% of a panel of 208 strains [71]. Hopefully, these discoveries can be translated into human studies in the near future.

## Compliance with ethics guidelines

Qian Wang and Linqi Zhang declare that they have no conflict of interest. This manuscript is a review article and does not involve a research protocol requiring approval by the relevant institutional review board or ethics committee.

**Open Access** This article is licensed under a Creative Commons Attribution 4.0 International License, which permits use, sharing, adaptation, distribution and reproduction in any medium or format, as long as you give appropriate credit to the original author(s) and the source, provide a link to the Creative Commons licence, and indicate if changes were made.

The images or other third party material in this article are included in the article's Creative Commons licence, unless indicated otherwise in a credit line to the material. If material is not included in the article's Creative Commons licence and your intended use is not permitted by statutory regulation or exceeds the permitted use, you will need to obtain permission directly from the copyright holder.

To view a copy of this licence, visit https://creativecommons.org/licenses/by/4.0/.
